# Effects of Transmission Delay on Client Participation in Video-Mediated Group Health Counseling

**DOI:** 10.1177/10497323211010726

**Published:** 2021-05-20

**Authors:** Sakari Ilomäki, Johanna Ruusuvuori, Jaana Laitinen

**Affiliations:** 1 Tampere University, Tampere, Finland; 2 Finnish Institute of Occupational Health, Helsinki, Finland

**Keywords:** group counseling, client participation, social processes, video-mediated interaction, conversation analysis, Finland, qualitative

## Abstract

In face-to-face group counseling, active client participation contributes to the counseling agenda by a variety of social processes, but little is known about how video mediation shapes client participation. In this article, we use conversation analysis to investigate how transmission delay affects client participation in video-mediated group counseling through shaping the resolution of overlapping talk. Data are video recordings from three video-mediated group health counseling sessions recorded simultaneously in the two participating locations. The delay changes the timing of the overlapping turns and pauses at each end of the mediated counseling, making it difficult to interpret who should take the turn after the overlap. This may pose obstacles to client participation. While mediated counseling services can increase access to services and thus improve client participation at a macro level, transmission delay can pose threats to active client participation at the micro level of interaction.

Group health counseling offers practical knowledge about healthier living and enables beneficial social processes that are not available in individual counseling, such as social support (Cormack et al., 2018; Frigerio & Montali, 2016; Logren et al., 2019a, 2019b). With digital technologies having become widespread, counseling can be provided online, as through synchronous video-mediated (VM) encounters in which counseling clients and supervisors meet in a video conference. In addition to its effectiveness in, for instance, diabetes care (Laitinen et al., 2010), VM counseling has many other potential benefits, such as increased access to services, especially in peripheral areas, and service provision without physical contact during epidemics like COVID-19. Problems with communication are often pointed out as deficiencies of both text-based counseling (Dilkes-Frayne et al., 2019; Stommel & van der Houwen, 2014) and VM interaction in other health care settings (Dalley et al., 2021), but less is known about how VM shapes the interactional dynamics of participation in group counseling. In this article, we examine how interaction dynamics, possibilities for client participation, and related positive social processes in VM group health counseling for Type 2 diabetes are shaped by transmission delay caused by the technical processes of VM. Type 2 diabetes was selected as it is the most common type of diabetes and its onset can be reduced by lifestyle changes (Gomersall et al., 2011; Ingadottir & Halldorsdottir, 2008; Knutsen et al., 2017; Rosenbek Minet et al., 2011; World Health Organization, 2020).

A central aspect of client participation is engagement in various kinds of activities (Castro et al., 2016; Halabi et al., 2020). Engagement can be examined at the level of both attending to counseling programs or interventions and the social dynamics of participation in counseling interaction. Low health literacy, stigma, gaps between the content offered and client lifeworld and needs, and externalized motivation have all been recognized as barriers to participating in diabetes counseling (e.g., Harris et al., 2019; Kinnafick et al., 2014; Vega et al., 2014). It has been suggested that feelings of stigma, irrelevance of content, and externalized motivation are all particularly suitable to being alleviated by group counseling, which aims at reflection and finding solutions and strategies, together with peers, that fit clients’ different life situations (Leong, 2008; Logren et al., 2017b). Furthermore, when people do engage in peer counseling, group-oriented activities and social support have been emphasized as benefits (Frigerio & Montali, 2016; Mendenhall et al., 2012). In sum, previous research maintains that active participation at the level of interaction dynamics is a prerequisite for effective group counseling: Such positive social dynamics can only emerge when counseling clients interact with one another and the supervisor (e.g., Hughes et al., 2017; Taggart et al., 2012; see Peräkylä & Ruusuvuori, 2007, for client participation as an interactional phenomenon in other health care contexts).

A growing strand of research on interaction processes has emerged to investigate the practices and processes that enable participation in different counseling settings (e.g., Miller & Silverman, 1995). These studies have described both ways in which professionals can encourage participation and how clients are able to initiate an active role in counseling encounters. For example, a counseling format that revolves around instructors’ questions and clients’ answers can limit client participation to merely answering questions in either individual or group counseling (Karhila et al., 2003; Logren et al., 2017a, 2017b; Poskiparta et al., 1998, 2001; Tiitinen et al., 2018). Furthermore, Karhila et al. (2003) have shown how direct presentation of troublesome issues in clients’ lives invites discussion about their lifestyle while merely hinting at possible problems may invite only minimal participation. Logren et al. (2017a) have described how clients can ask questions to produce shifts from leader-driven to member-driven discussion, which then affords increased client participation and exchange of experiences among peers. By responding to one another’s experiences, clients can provide social support, offer different perspectives on the topic at hand, and challenge other clients’ thinking in constructive ways (Logren et al., 2017b, 2019a, 2019b). When group members engage in these practices, they work collaboratively toward the goals of counseling, sometimes in ways that are not available to the counselor. While these previous studies have provided important knowledge of the practices and processes through which participation is enabled in copresent counseling situations, detailed study of such practices in VM settings and how the VM setting affects them are lacking.

VM settings demand new kinds of interactional competence from professionals because of the limitations of the medium (Dalley et al., 2021). One omnipresent limitation of VM interaction is transmission delay, which is the difference between the point in time when a participant says something and when the other participants hear it, a delay caused by the technical processes of transmission (Schoenenberg et al., 2014). It has been shown that transmission delay can complicate turn-taking and therefore cause interactional dysfluency and a lack of awareness regarding the rights to speak, such as unintended pauses and overlapping talk (Ruhleder & Jordan, 2001; Rusk & Pörn, 2019; Seuren et al., 2020). In their recent study on VM consultations between individual patients and their doctors, Seuren et al. (2020) showed that transmission delay can cause problems with turn-taking and produces unintended interruptions and silences.

In this study, we explore how VM affects the interaction dynamics in group counseling contexts and how such effects shape the emergence of positive social processes of group counseling. We focus on examining how the nutritionist leading the group and the group members resolve overlapping talk (Schegloff, 2000) in VM group counseling and what kind of client participation emerges. Our overall aim is to reveal the potential risks that VM may pose to active participation in counseling and to suggest ways to overcome these problems.

## Data and Method

The data are video recordings of three VM health counseling sessions for adults at increased risk for Type 2 diabetes. We conducted a secondary analysis of these data, which were originally used for studying the feasibility of VM counseling, features of behavior change, and motivational factors of health behavior (Laitinen et al., 2010; see also Alahuhta et al., 2011; Korkiakangas et al., 2010; Nevanperä et al., 2015). Nurses and doctors working in occupational and primary health care recruited the clients. The clients were eligible to participate if they were at high risk for Type 2 diabetes, did not have any ongoing serious illness, did not use medication to treat obesity, and were not on a very low-calorie diet. VM counseling was offered to people living in municipalities where face-to-face counseling was not available (from 40 to 91 km away from the regional center). At the time of the data collection (2006), secure videoconferencing systems were not widespread, so the group gathered in a local health care service unit and connected with the nutritionist via video link. There were no professionals in the room during the meetings, but local nurses helped with setting up the system, and group members could contact them if any problems arose. Despite being from the early 2000s, the data afforded a fruitful analysis (see “Discussion” section for further commentary on the limitations of the data).

The group counseling intervention aimed at promoting learning and a process of change. The overall goals of the counseling were to become aware of diabetes-related lifestyle habits and their importance in preventing diabetes, to learn new skills for a healthier life, to normalize eating behavior, and to exercise regularly. The groups met four times fortnightly and a fifth time 6 months after the fourth session. Each session lasted approximately 1½ hours and followed a designated structure of activities and topics. Group activities included mindfulness-like attuning, discussing the homework given in the last sessions (e.g., keeping a food diary) and the topic of the meeting, and playing a board game with diabetes-relevant information and scenario-based dilemmas. Further information about the groups is presented in Table 1; for more detail, see Laitinen et al. (2010).

**Table 1.** Group Characteristics.

Group Number	Meeting Number	Summary of Meeting Content	Group Members	Nutritionist	Number of Sequences Analyzed (*N* = 79)
1	1/5	Overview, methods, and rules of the group. Reflecting on current weight management and health.	8 females	A	34
2	3/5	Healthy eating.	7 females	B	22
3	5/5	Reflecting on current weight management and health. Imagining future.	4 males	A	23

The counseling intervention was developed and delivered by the Finnish Institute of Occupational Health. Jaana Laitinen was part of the team that oversaw the intervention and organized the data collection. Sakari Ilomäki and Johanna Ruusuvuori conducted the analysis and did not participate in organizing the intervention. Informed consent to participate in the study was obtained from all participants. The coordinating ethics committee of the hospital district of Helsinki and Uusimaa (Document Number 50/E0/2007) approved the study.

The counseling sessions were video recorded from both the nutritionists’ and the groups’ perspectives; that is, each encounter was simultaneously recorded at the nutritionist’s office and in the room where the group met. This enabled us to study how the interaction unfolded differently when perceived from the nutritionist’s perspective in relation to the group’s perspective (we refer to this difference in perspectives as *non-mutual interactional realities*; see Ruhleder & Jordan, 2001). These three sessions were selected from a larger corpus of 21 VM sessions, as they were the only ones with recordings from both perspectives. The availability of the two separates but simultaneous perspectives created a unique opportunity to explore how transmission delay can shape interaction. We transcribed the recordings using the Jeffersonian transcription system (Jefferson, 2004a: see the supplementary materials for transcription symbols). In the extracts, we present data from both perspectives and refer to the line numbering of different transcripts by using the letters G (the group’s perspective) and N (the nutritionist’s perspective). The original data are in Finnish, but the extracts are in English (see the supplementary material for extracts with Finnish data).

We used conversation analysis (CA), an inductive method for examining the practices and structures of interaction from the participants’ viewpoint (e.g., Sidnell & Stivers, 2013). In CA, attention is paid to how turns are formulated, how participants manage speaker change, and how they make sense of the ongoing interaction turn by turn. We focused on the instances that started with the nutritionist’s counseling-relevant questions and contained overlapping talk (*N*=79). We selected the nutritionist’s questions as the starting point because of their importance in directing counseling interaction (e.g., Logren et al., 2017a; Poskiparta et al., 1998). We analyzed how the participants decide who gets to talk after overlapping talk in these segments (Schegloff, 2000). By focusing on how overlaps are resolved, we aimed to analyze situations that would be of particular importance for the direction that the interaction takes and thus more prone to the effects of technical mediation. We paid special attention to the location of the overlapping turns and the implications that these locations have on interaction (see Drew, 2009). The ways of resolving overlaps were then analyzed from the viewpoint of how the participants’ different perspectives affect the overlap resolution. Finally, we reflected on how these differences shaped the clients’ possibilities of participating in the counseling discourse.

Before focusing on the effect of VM on counseling interaction, we briefly show how overlap resolution takes place in face-to-face counseling. The group has been discussing a healthy diet (Extract from Logren et al., 2017a, p. 1837). N refers to the nutritionist, while A, B, and C are group members.

Extract 1: Overlap resolution in face-to-face situation

 1 A: Isn’t banana fa:ttening.
 2   (1.2)
 3 **N: No it [its**
 4 **B: [Don’t** throw it out right away **[wh(h)en ( )** hah hah hah
 5 N:  **[Weh heh heh**
 6 N: **I[:t,**
 7 A: **[One hears** all **kinds[of things.**
 8 N:  **[It is,**
 9 C:  Mm
10 N: ↑like a belief that is (.) terribly strong.

Extract 1 exemplifies four issues that are typical of overlapping talk in face-to-face or co-present interaction. First, overlapping talk occurs frequently and usually lasts for only short periods (Sacks et al., 1974). Second, the participants monitor the ongoing stretch of talk to deduce when it is appropriate to take their turn (Goodwin & Goodwin, 2004). For example, in lines 4 and 5 the nurse starts laughing immediately when B’s turn is understandable or, in CA-terms, reaches a transition-relevance place (Sacks et al., 1974, p. 704), resulting in an overlap with the last word spoken by B. Third, when facing overlaps, the participants solve them beat by beat—word by word or even syllable by syllable—until only one person is talking (Schegloff, 2000). Fourth, the participants use the timing of the overlap as a hint for speaker selection (Drew, 2009; Jefferson, 2004b). Overlaps occurring after the transition relevance place may result in giving one’s turn to the individual initiating the overlap (as the nutritionist interrupts her turns when group members start talking in lines 4 and 7), while overlaps occurring close to the end of another participant’s turn are treated as legitimate, not resulting in such ruptures (like the nutritionist’s overlapping turns near the closure of group member’s turns, in lines 5 and 8). In all, resolving overlapping talk is closely tied to the timing of turns. Next, we show what happens when this timing is complicated by the delay that occurs in VM counseling.

## Results

Transmission delay changes the timing of the turns and overlaps and affects the different ways of resolving overlap such as timing of the pauses within the turn. This leads to different interpretations of who should talk after the overlap, which affects the group members’ possibilities of participating in three ways. First, unintended overlaps and uncertainty about the next speaker can lead to competition over the turn when activity is about to change (Extract 2). Second, perceived silences in the nutritionist’s talk, which in face-to-face conversation would offer the turn to the client, may be treated as pauses within the nutritionist’s turn and not as turn offerings (Extract 3). Third, to secure the speakership of the client, the nutritionists can offer the turn explicitly by asking the client to speak (Extract 4).

### The Delay Produces Different Orientations to the Progression of Interaction, Which Leads to Competition Over the Turn

Changes in the appearances of turns, overlaps, and ways of resolving overlaps lead to different interpretations of the ongoing activity in each location of the VM encounter. This is exemplified in Extract 2, where delay-generated changes lead to different understandings about the relevance of a topic change and the need for overlap resolution. Before Extract 2, the group was doing an exercise in which they wrote down something they had already succeeded at, and the nutritionist then asked everybody to read their answers. Group member A said that she has switched from candy to dried fruit. The extracts illustrate the discussion during the same time slot, as it appears to the participants in the two different locations, showing first the group’s perspective and then the nutritionist’s perspective.

Extract 2

### Group’s Perspective (Group 2)

1 N: Has it been <hard> to walk past that candy [shelf.]
 2 A: [No i- ] (.) it hasn’t
3   been hard when one has made that decision that °I’m not going
 4 A: there anymore [   (-]-)°
 5 ?: [°.hhm° ]
 6   (0.4)
 7 N: °↓Yes.°
8   (1.1)
9 N: **.hh[hh ]  [ (Eating)  sweets-    ]**
10 A: **[ Bu]t th[at craving- (.) ↑CRAVING FO]R SWEETS** where does it
11   come from.

   ((Continues))

### Nutritionist’s Perspective

 1 N: Has it been HARD to walk past that candy shelf.
 2   (0.7)
 3 A: No i- (.) ↑it hasn’t been hard when one has made that decision
 4 A: °(that I’m not?)°
 5   (0.3)
 6 N: Yes:.
 7   (1.3)
 8 N: **.hhhh EATING swe[ets-    ]**
9 A:  **[(-ut that cra]ving,** (.) craving for sweets >where
10   does it< come from.

 ((Continues))

The extracts show how transmission delay causes the occurrence of overlapping talk to be situated differently in each location. While the overlap does not happen until in lines 9–10G/8–9N, the preceding interaction lays the ground for different interpretations of the overlap. From the *group’s perspective*, it seems that following the nutritionist’s question and A’s answer, group members have an opportunity to take their turn (line 10G), as there is a gap of 1.1 seconds (line 8G/line 7N) and the nutritionist has not yet started her turn. But from the *nutritionist’s perspective*, it seems that the interactants are ready to move on to the next activity (Heritage & Sefi, 1992), so she starts her turn (line 8N). When she hears group member A, who seems to have started later, or in CA terms in *interjacent overlap* (line 9N, Drew, 2009, 88–91), she makes the choice to abandon her turn to implicitly offer it to group member A. This temporally caused confusion in terms of the progression of the discussion results in the group member starting to compete over the turn with the nutritionist. This is shown in her continuing to talk in spite of hearing the nutritionist also starting to talk, and in speaking louder to maintain the turn (lines 9–10G/8–9N). From the nutritionist’s perspective, however, as the group member starts clearly later than she does, she is not competing over the turn but gives it to the group member (who has seemingly interrupted her, line 8–9N). A’s competition over the turn and the nutritionist’s dropping out enable A to participate by posing her topic-relevant question, thus producing a shift from leader-driven to member-driven discussion (Logren et al., 2017a).

Due to delay-generated changes, the participants perceive the overlapping talk and its implications for interaction differently: Competition over the turn appears relevant to A, while competition is not relevant for the nutritionist. In this case, the delay and the changes it generated in the position of the overlap eventually helped A to take the turn despite the lack of shared perspective on interaction, but as the following extracts show, these changes can also prevent client participation.

### Implicit Turn-Offering Is Perceived as a Within-Turn Pause

When the participants implicitly offer the turn to one another by pausing, the transmission delay can alter the location of the pause and make it appear as a pause within the speaker’s turn. This kind of pause does not unambiguously afford speaker change and participation (Sacks et al., 1974). Furthermore, as was exemplified with the nutritionist abandoning her turn in the previous extract, when participants find themselves in interjacent overlap, the position of the overlap hints that there are some reasons for the other participant to continue and for the current speaker to stop (Drew, 2009, pp. 88–91). These two dynamics are at play in the following extract, where the nutritionist offers the turn to group member C by pausing (line 34N), but the delay leads C to perceive the nutritionist’s turn as interjacent overlap and the turn-offering pause as occurring within the nutritionist’s ongoing turn (line 34G). Due to the changes in timing, the nutritionist’s turn-offering pause is apparent only in the second transcript (line 34N), not in the first (line 34G). The group is discussing their challenges regarding eating.

Extract 3

### Group’s Perspective (Group 2)

 1 N: .hhhhhhh Well how about A?
 2   (0.3)
 3 N: What do you have.=
4 A: =Well I have a point exact[ly about this?] (0.2) kr kr krhm (0.8)
 5 ?:   [ .nffft   ]
6 A: this evening- evening tiredness and that when I take supper so,
 ((16 lines omitted. A describes her difficulties in managing eating in the evenings. B responds by telling how she prepares the number of sandwiches she is about to eat and then puts the ingredients away to avoid overeating.))
22 B: .hhh Heh[ heh£ ]  [ .hhh ]
23 A:  [°Yeah.°] So [it’s like] that, (.) my problem that I eat
24   more then >↑in the evening<=I don’t like during the day I don’t
25   have problems and neither then still when I come from work so? .hhh
26   there is no problem with eating but the supper is kind of a, (0.3)
27 A: < stumbling [°block.°> ]
28 B:  [°(I have <exactly] the same.) (—)°
29   (0.4)
30 N: Ye:s.=
31 B: =°In principle.°
32   (0.4)
33 C: **Yeah and then [that:,]**
34 N:      **[ Rea]lly good, (0.7) really good suggestion,**

 ((Continues)) ((N refers to B’s suggestion which is not shown.))

### Nutritionist’s Perspective

 1 N: .hhhhhhh Well how about A?
2   (0.3)
3 N: What do you have.
 4   (1.3)
 5 A: Well I have a point exact[ly about this?] (0.2) kr kr krhm (0.8)
 6 ?:  [ .nffft   ]
 7 A: this evening- evening tiredness and that when I take supper so,
   ((16 lines omitted.))
22 B: .hhh heh[ heh£ ]  [ .hhh ]
23 A:  [°Yeah.°] So [it’s like] that, (.) my problem that I eat
24   more then >↑in the evening<=I don’t like during the day I don’t
25   have problems and neither then still when I come from work so?
26   .hhh (0.2) there is no problem with eating but the supper is kind
27   of a, (0.3) stumbling °block.°
28   (1.1)
29 N: Ye:s.
30   (1.0)
31 B: °In principle.°=
32 N: **=Reall[y good m? ]**
33 C:  **[Yeah (—-) ]**
34   **(0.7)**
35 N: **Really good suggestion,**

 ((Continues))

Again, what happens before the overlap (lines 33–34G/32–35N) lays the ground for the different interpretations of participation. After the nutritionist’s question and A’s answer, A and group member B start discussing a possible solution to A’s problem. A’s repetition of the central point of her answer (lines 23–27G/23–27N; see, for example, Barnes, 2007), B’s statement of sharing the same experience (line 28G/not hearable by the nutritionist; Logren et al., 2019a), and brief turns by the nutritionist and B (lines 30–31G/29, 31N; Schegloff, 2007) all imply that they are finished with the previous topic and ready move on in the discussion. From the *nutritionist’s perspective*, she starts to evaluate the idea, which was discussed earlier, of how to avoid evening snacking (line 32N) but stops immediately when she hears C’s voice and implicitly offers the turn to her by pausing (lines 33–34N). As C does not appear to continue, the nutritionist finishes the evaluation and repeats the advice provided by the group members (line 35N). From the *group’s perspective*, the implicit turn offering appears a bit differently due to the alterations that the delay causes on the location of the overlap and the nutritionist’s pause. The third group member, C, starts adding a new perspective to the topic (“yeah and then that,” line 33G) but finds herself in an interjacent overlap with the nutritionist (line 34G). Like the nutritionist in Extract 2, C takes this overlap to imply that the nutritionist should take the turn and stops talking. From the group’s perspective, the pause (with which the nutritionist, from her perspective, implicitly offered a turn to C) appears as a pause within the nutritionist’s turn, during which speaker change is not relevant.

In Extract 3, the interactional implications of the overlapping talk and turn-offering pause appear to be different in the different locations because of the non-mutual interactional realities. From the *nutritionist’s perspective*, she has provided C with a reasonable amount of time to take the turn, but from the *group’s perspective*, the pause that was supposed to offer the turn occurred in a location that did not make the speaker change relevant. The comparison of the two perspectives reveals a central shortcoming of implicitly offering the turn in overlap situations during VM counseling: Technological mediation produces a situation where implicit turn offerings may appear as pauses within the leader’s turn and thus be inadequate to secure client participation. Furthermore, unlike Extract 2, where A meets the nutritionist’s overlapping talk in a similar interactional environment and competes over the turn, C does not engage in competition over the turn here. This might show her orientation to herself as not a ratified speaker at this point, as she is not the one to whom the question was posed (as A was in Extract 2). Whatever the reason, since C does not engage in competition and the nutritionist’s implicit turn offering is ineffective to secure the turn for C, her participation in the discussion by responding to another group member’s challenging experience (Logren et al., 2019a) and related positive social contribution are not actualized.

### Explicit Turn Offering Secures Client Participation

When the nutritionist recognizes that her implicit turn offering has fallen short, the turn can be offered more explicitly with minimal offerings (such as “yeah” with a rising intonation and other continuers; Müller, 1996) or more direct offerings like “go ahead.” As in co-present interaction, explicitly offering the turn secures client participation as it indicates that the one who offered the turn has a right—and in some sense an obligation—to take the turn (Sacks et al., 1974). This is exemplified in Extract 4. The group is playing a boardgame that involves information about healthy lifestyle, questions about diabetes, and scenario-based dilemmas to solve. The group member A has been given the task of naming three good practices of stress management and she has suggested physical exercise, handicrafts, and reading.

Extract 4

### Group’s Perspective (Group 2)

 1 N: Really good tips.
 2 N: You got three points.
 3 N: [Does somebody want] to add something more, (.) to this issue.
 4 ?: [  ↑mm:?   ]
 5   (4.1)
 6 B: **Well=nothing more than [ that-  ]**
 7 N: ** [As we know] stress affect- (.)** ↑**yeah?**
 8 B: **Th[at-  (.)  e-] I was thinking that when=it feels really**
 9 N: ** [>Go ahead.<]**
10 B: >stressful,< (0.4) I at least try to come up with something
11   to do >that I< like.
12   (1.0) ((Continues))

### Nutritionist’s Perspective

1 N: Really good tips.
 2 N: You got three points.
3 N: Does somebody want to [add some]thing more, (.) to this issue.
 4 ?:  [  (-)  ]
 5   (4.7)
 6 N: **As we kn[ow stress affect-   ]**
 7 B: ** [Well=nothing more than,]**
 8   **(.)**
 9 N: ↑**yeah?**
10   **(0.5)**
11 N: **>Go ahead.<**
12 B: **That- (.) e- I was thinking that when=it feels really**
13 B: **>stressful,<** (0.4) I at least try to come up with something to do
14 B: >that I< like.

 ((Continues))

After the nutritionist has commented on A’s ideas for stress management, she checks whether anybody has something to add (line 3G/3N). The question is not directed to a specific group member and not answering would be taken as the group’s readiness to move on to the next activity. From the *nutritionist’s perspective*, the long pause (line 5N) hints that nobody has anything to add, and she moves on to providing information about the interconnectedness of stress and eating (line 6N). From the *group’s perspective*, however, group member B starts her answer after a lengthy pause (line 3G) but finds herself interrupted by the nutritionist’s turn (line 4G). Like group member C in Extract 3, B ceases to talk when facing this kind of interjacent overlap and does not take the turn during the pause in the nutritionist’s turn (line 7G), since, from the group’s perspective, the turn-offering pause appears as a pause within the nutritionist’s turn. But, unlike in Extract 3, the nutritionist explicitly asks B to continue (“yeah,” lines 7G/9N). From the *group’s perspective*, B restarts her answer immediately after the explicit offer to take the turn, while from the *nutritionist’s perspective* the nutritionist experiences a half-second pause and further expands the offering with ”go ahead“ (lines 8/10–11 N). By building on the implicit turn offering with an explicit offer, the nutritionist enables B to take the turn and thus participate by a self-reflective turn (Logren et al., 2017b). In her response, B both expresses sharing the experience of stress management and reframes A’s list of potential practices of stress management at a more general level, thus helping others to identify practices that could fit their lifestyle and situation. Had the nutritionist not been sensitive to B’s turn and offered the turn explicitly, reflection and reframing would likely not have occurred.

## Discussion

We have shown how transmission delay shapes perceived rights to take the turn and thus client participation in VM group counseling, where the group meets in a single location to connect with the nutritionist via video link. The transmission delay caused by VM produced changes in the timing of the overlaps and the ways of resolving overlapping talk resulted in confusion in terms of speaker choice. Due to the delay, the group and the nutritionist had slightly different perspectives concerning the ongoing action; they had *non-mutual interactional realities* (Ruhleder & Jordan, 2001) that led to different interpretations of the progression of the discussion and which speaker rights would be timely in the moments of delay. This limited the group members’ possibility to participate. To secure their participation, the group members competed over the turn, especially in interactional intersections where the topic or broader line of action was changing and where the turn allocation was thus not explicitly determined. The nutritionists worked toward securing client participation by offering the turn implicitly by pausing or explicitly asking the client to continue. As Extract 3 shows, implicit turn offerings often proved to be inadequate to secure a speaker change and client participation. To overcome this problem, nutritionists could strive to offer turns explicitly. Compared with implicit turn offerings, explicit offerings were more likely to secure the client speakership and participation.

The analysis has revealed that, while the effect of transmission delay is apparent for the analyst and the readers of this article—who have data from both perspectives—it may have remained hidden from the participants. Technological aspects were not mentioned in meta-talk when facing overlapping talk but only when some of the participants could not produce a relevant next action (e.g., the nutritionist could not evaluate the client’s inaudible answer). These cases were rare, and the delay was never explicitly mentioned as a source of trouble. In addition, as implicit turn offering recurrently failed to secure a speaker change, it is plausible to suggest that the participants were not aware that the delay caused such drastic barriers to participation.

Our findings align with earlier research that has highlighted communication difficulties as the central problem of technologically mediated counseling (Dilkes-Frayne et al., 2019; Stommel & van der Houwen, 2014), VM interaction in health care (Dalley et al., 2021), and VM interaction in general (Ruhleder & Jordan, 2001; Rusk & Pörn, 2019). We add to this knowledge by showing that these difficulties may be difficult to notice and name and therefore shape the interaction even when they remain hidden. Our findings are in line with Seuren et al. (2020) who showed that delay interferes with regular turn-taking in VM consultations. We expand on this finding by showing that these interferences have consequences for client participation and beneficial social processes of counseling, such as peer support. To our knowledge, this topic has not been studied in any kind of group setting before.

Earlier research on interaction dynamics in counseling settings has demonstrated how clients’ possibilities are shaped on at least two intertwined levels: The lines of activities put forward in the interaction and how single turns of talk are designed to encourage different kinds of participation (Karhila et al., 2003; Logren et al., 2017a, 2017b, 2019a, 2019b; Poskiparta et al., 1998, 2001; Tiitinen et al., 2018). Our analysis showed that participation dynamics is also managed at the level of speakership after overlaps. Since resolving overlaps demands meticulous monitoring of the timing of overlapping turns and pauses, this level is heavily influenced by technological mediation, potentially even more than the other two. Furthermore, these ruptures, in participation, occur in relation to activities that are central to fulfilling the positive social processes in counseling such as steering the direction of interaction to member-led discussion (Extract 2; Logren et al., 2017a) and responding to other members’ turns in constructive ways (Extracts 3 and 4; Logren et al., 2019a). In all, while VM counseling services can increase access to services and thus improve client participation at the macro level, transmission delay caused by the technology involved can pose threats to active client participation and the associated positive social dynamics at the micro-level of interaction.

The existing qualitative research on patient and client participation has predominantly concentrated on experiences of participation (e.g., Gomersall et al., 2011). Considering participation as an ongoing interactional achievement (Collins et al., 2007; Goodwin & Goodwin, 2004) offers important insights into the conceptualization and study of participation by providing a more nuanced understanding of how engagement in counseling activities evolves in situ. The interactional perspective contributes to understanding how the working relationship and engagement in activities that contribute to the goals of the counseling unfold in discourse and how technological mediation shapes the possibilities to do so. These two perspectives—client experience and interaction dynamics—are not conflicting but complementary (De Jaeger et al., 2016). Connecting the study of interaction dynamics and clients’ and professionals’ experiences could be strengthened in the future to form a more holistic understanding of participation in VM counseling.

To understand the ways in which technological mediation shapes interaction dynamics in different health care settings more deeply, further research in at least two areas is needed. First is how the features of the specific health setting shape ways of participating: for example, is participation in VM counseling, which aims at reflection and problem solving, different from tele-consultations, which aim at diagnosis and decision-making about the treatment, and are there differences between patient groups and illnesses? Second is how participation is managed in technologically mediated interactions with different numbers of participants and sites of interaction. As troubles for client participation arose even with only two sites of interaction, we hypothesize that managing participation in settings with three or more sites would be even more complex.

The use of VM counseling and other health services is likely to continue to grow in the future. Urbanization and the decay of peripheries, the ideals of aging in place and, perhaps most pressingly, recurring pandemics that demand refraining from physical contact will increase the use of VM services. Simultaneously, Type 2 diabetes and other chronic conditions that could be alleviated with proper lifestyles are becoming more common globally. If high-quality care and counseling are to be offered through VM, it is crucial to increase our understanding of how different features of the technology affect participation, working relationships, and engagement.

## Conclusion

### Strengths and Limitations of the Study

The small dataset of this study was based on necessity: We used all the data that the project had gathered from two perspectives of action. This is a clear strength, as our findings regarding the non-mutual interactional realities would have been impossible to make without this kind of rich data. While the small dataset could limit the generalizability of our findings, CA of institutional encounters aims not only at finding generalizable practices but also at describing what kind of practices are possible in a specific context (Peräkylä, 2004). In this research model, generalizability stems from comparing the findings from different contexts, which in our case means earlier findings from the counseling context (Karhila et al., 2003; Logren et al., 2017a, 2017b, 2019a, 2019b; Poskiparta et al., 1998, 2001) and research on VM interaction in different contexts (Ruhleder & Jordan, 2001; Rusk & Pörn, 2019; Seuren et al., 2020). Moreover, by using the concept of participation (Castro et al., 2016; Collins et al., 2007; Halabi et al., 2020) as a basis for theoretical comparison, we have been able to show how delay, which is a general feature of any kind of VM interaction, becomes consequential specifically in counseling contexts. This has enabled us to participate in discussions beyond our empirical cases. The fact that the data were gathered approximately 15 years ago could be considered as a limitation to its validity. However, despite 15 years of technological development and improved tele-health competencies, the delay-generated problems that we described are present in more recently gathered data as well (see Seuren et al., 2020), thus justifying the secondary analysis of data.

### Implications for Practice

Video mediation is a challenging environment for nutritionists and other professionals to lead peer groups. In general, we hope to have sparked reflection among health care professionals about the effects that VM has on interaction and client participation. As the analysis has shown, the methods used in face-to-face interaction for securing client participation after overlaps may be inadequate in a VM setting. To secure client participation and positive social processes, the nutritionists in our data offered explicitly the turn to participants. Even though offering the turn explicitly can, in some cases, result in new overlaps or awkward feeling, our data suggest that it is more reliable for ensuring client participation after overlaps than implicit turn offerings. Furthermore, meta-talk about the effects of technical mediation on the interaction process and working relationship might alleviate the possible negative feelings associated with overlaps and confusion concerning speaker choice. Our findings about the fine details of interaction and their relationship with client participation might have remained unnoticed if the participants were merely interviewed about the quality of interaction. Therefore, we encourage practitioners and researchers to engage in studies that draw data from real-life health care interactions and pay attention primarily to interaction dynamics as an endogenous phenomenon.

## Supplemental Material

sj-pdf-1-qhr-10.1177_10497323211010726 – Supplemental material for Effects of Transmission Delay on Client Participation in Video-Mediated Group Health Counseling Click here for additional data file. Supplemental material, sj-pdf-1-qhr-10.1177_10497323211010726 for Effects of Transmission Delay on Client Participation in Video-Mediated Group Health Counseling by Sakari Ilomäki, Johanna Ruusuvuori and Jaana Laitinen in Qualitative Health Research

sj-pdf-2-qhr-10.1177_10497323211010726 – Supplemental material for Effects of Transmission Delay on Client Participation in Video-Mediated Group Health Counseling Click here for additional data file. Supplemental material, sj-pdf-2-qhr-10.1177_10497323211010726 for Effects of Transmission Delay on Client Participation in Video-Mediated Group Health Counseling by Sakari Ilomäki, Johanna Ruusuvuori and Jaana Laitinen in Qualitative Health Research
