# Supplementary material for: Effects of Transmission Delay on Client Participation in Video-Mediated Group Health Counseling
Source: Qual Health Res. 2021 May 20;31(12):2328–39. doi: 10.1177/10497323211010726 (PMC8564242; doi:10.1177/10497323211010726)
Supplement: sj-pdf-1-qhr-10.1177_10497323211010726 – Supplemental material for Effects of Transmission Delay on Client Participation in Video-Mediated Group Health Counseling [file sj-pdf-1-qhr-10.1177_10497323211010726.pdf]

Supplementary material 1: Transcription symbols

[word]	Brackets: onset and offset of overlapping talk
=	Equals sign: contiguous utterances, second is latched immediately onto the first
(0.2)	Timed interval within or between utterances, measured in seconds and tenths of seconds
(.)	Interval of less than 0.2 s
wo:rd	Colon: extension of the sound or syllable
.	Full stop: falling intonation
,	Comma: continuing intonation
?	Question mark: rising intonation1
↑	Upward arrow: Rising pitch
wo-	Dash: abrupt cut-off
WORD	Capital letters: louder volume
<word>	Slower-paced talk than the surrounding talk
>word<	Faster-paced talk than the surrounding talk
°word°	Degree signs: quieter volume
#word#	Hash sign: squeaky voice
hh	Audible aspiration.
hh	Audible inhalation
w(h)ord	Laughter
(----)	Lines in paranthesis: Unclear and unidentifiable talk
((word))	Text in parentheses: transcriber’s comments
Q	question word or particle used in glossings
CLT	clitic
PTCL	particle
