# Supplementary material for: Effects of Transmission Delay on Client Participation in Video-Mediated Group Health Counseling
Source: Qual Health Res. 2021 May 20;31(12):2328–39. doi: 10.1177/10497323211010726 (PMC8564242; doi:10.1177/10497323211010726)
Supplement: sj-pdf-2-qhr-10.1177_10497323211010726 – Supplemental material for Effects of Transmission Delay on Client Participation in Video-Mediated Group Health Counseling [file sj-pdf-2-qhr-10.1177_10497323211010726.pdf]

Supplementary material 2: Data extracts with original Finnish transcripts and word-by-word glossing

Extract 1

1 A: **eikö banaani liho:ta.**
not+Q banana fatten
Isn't banana fa:ttening.
2 (1.2)
3 N: **ei se [sen**
No it [its
4 B: **[elä nyt heti tyrmää [k(h)u ( )hah hah hah**
don't+you now immediatelly knock+out when
[Don't throw it out right away [wh(h)en ( ) hah hah hah
5 N: [Weh heh heh
6 N: **s[e:,**
I[:t,
7 A: **[sitä kuulee kaiken[laista.**
it 0+hears all+kinds+of+things
[One hears all kinds[of things.
8 N: **[se on,**
[It is,
9 C: Mm
10 N: ↑**semmonen hirveen vahvassa, (.) oleva usko.**
the+kind+of, terribly strongly existing belief
↑like a belief that is, (.) terribly strong.

Extract 2

Group's perspective (Group 2)

1 N: **onko ollu <vaikeeta> kävellä sen karkkihyllyn o[hi.]**

has+Q been hard to+walk that candy+shelf past

has it been <hard> to walk past that candy [shelf.]

2 A: ***[ei s-] (.) ei oo***

no not has

[no i-] (.) it hasn't

3 **ollu vaikeeta kun sen tehny päätöksen että °ei ny (ruukaa)°**

been hard when that done decision that not now (have+habit+of)

been hard when one has made that decision that °I'm not going

4 A: *[(-]-)°*

there anymore [(-]-)°

5 ?: [°.hhm°]

6 (0.4)

7 N: °↓***joo.***°

yes

°↓yes.°

8 (1.1)

9 N:**.hh[hh] [makean (syöntihä-)]**

sweet's eating

.hh[hh] [(eating) sweets-]

10 A: **[mut]ta s[e makkeen- (.) ↑MAKKEEN HI]MO NI mistä se voipi**

but that sweet's sweet's graving like where it can

[bu]t th[at craving- (.) ↑CRAVING FO]R SWEETS where does it

11 **johtua.**

be+due

come from.

((Continues))

Nutritionist's perspective

1 N: **onko ollu VAIKEETA kävellä sen karkkihyllyn ohi.**

has+Q been hard to+walk that candy+shelf past

has it been HARD to walk past that candy shelf.

2 (0.7)

3 A: **ei s- (.) ↑ei oo ollu vaikeeta kun sen on tehny päätöksen**

no   not has been hard   when that has done decision

No i- (.) ↑it hasn’t been hard when one has made that decision

4 A: °(that I’m not?)°

°(ettei?)°

that+not

5 (0.3)

6 N: **joo:.**

yes

yes:.

7 (1.3)

8 N: **.hhh MAKEAN syön[tihä-   ]**

sweet’s eating

.hhhh   EATING   swe[ets-   ]

9 L: **[(-ta se   ma]kee,) (.) makeen himo   nii >mistä se<**

that   sweet’s   sweet’s graving like where it

[(-ut that cra]ving, (.) craving for sweets >where

10 **voipi johtua.**

can be+due

does it< come from.

((Continues))

Extract 3

Group's perspective (Group 2)

1 N: **.hhhhhhh no entäs A?**

well how+about A

.hhhhhhh well how about A?

2 (0.3)

3 N: **mitäs sulla ois.=**

what you would+have

what do you have.=

4 A: **=no mullon jus[t tähä?] (0.2) kr kr krhm (0.8)**

well I+have exactly to+this

=well I have a point exact[ly to this?] (0.2) kr kr krhm (0.8)

5 ???: [.nffft]

6 A: **tämä ilta- ilta(nen) väsymys ja se et kun iltapaa otan nii,**

this evening evening tiredness and it that when supper I+take so

this evening- evening tiredness and that when I take supper so,

((16 lines omitted. A tells about her difficulties in managing eating in the evenings. B responds by telling how she prepares the amount of sandwiches she is about to eat and then puts the ingredients away to avoid over eating.))

22 B: .hhh heh[heh£] [.hhh]

23 A: **[° nii.°] et [se on niinku] se, (.) minu' ongelma että syön**

yeah so it is like it my problem that I+eat

[°yeah.°] so [it's like] that, (.) my problem that I eat

24 **enemmän >↑illalla<=mulle ei niinku päivällä mullei**

more in+evening to+me no like during+day I+don't

more then >↑in the evening<=I don't like during the day I don't

25 **oo ongelmaa eikä oo sillonkaa vielä ku mä töistä mee' niin? .hhh**

have problem neither have then+even still when I from+work go so

have problems and neither then still when I come from work so? .hhh

26 **siinä syönnis ei oo ongelmaa mut se iltapala on semmone, (0.3)**

in+that eating no is problem but that supper is that+kind+of

there is no problem with eating but the supper is kind of a, (0.3)

27 A: **< kompastus[°kivi.°>]**

stumbling+block

< stumbling [°block.°>]

28 B: **[°(mullon <aivan] samallailla.) (--)°**

I+have exactly same+way

[°(I have <exactly] the same.) (--)°

29 (0.4)

30 N: **joo:.=**

yes

ye:s.=

31 B: **=°ajatuksen tasolla.**

thought's level

=°in principle.°

32 (0.4)

33 C: **nii ja sitte [se:,]**

yeah and then that

yeah and then [that:,]

34 N: **[hirv]eän hyvä, (0.7) hirveän hyvä ehdotus,**

awfully good awfully good suggestion

[rea]lly good, (0.7) really good suggestion,

((Continues))

Nutritionist's perspective

1 N: **.hhhhhhh no entäs A?**

well how+about+s A

.hhhhhhh well how about A?

2 (0.3)

3 N: **mitäs sulla ois.**

what you would+have

what do you have.

4 (1.3)

5 A: **no mullon jus[t tähä?] (0.2) kr kr krhm (0.8)**

well I+have exactly to+this

well I have a point exact[ly to this?] (0.2) kr kr krhm (0.8)

6 ?: [.nffft]

7 A: **tämä ilta- ilta(nen) väsymys ja se et kun iltapaa otan nii,**

this evening evening tiredness and it that when supper I+take so

this evening- evening tiredness and that when I take supper so,

((16 lines omitted. A tells about her difficulties in managing eating in the evenings. B responds by telling how she prepares the amount of sandwiches she is about to eat and then puts the ingredients away to avoid over eating.))

22 B: .hhh heh[heh£] [.hhh]

23 A: **[°nii.°] et [se on niinku] se, (.) minu' ongelma että syön**

yeah so it is like it my problem that I+eat

[°yeah.°] so [it’s like] that, (.) my problem that I eat

24 **enemmän sillon >↑illalla<=mulle ei niinku päivällä mullei**

more then in+evening to+me no like during+day I+don’t

more then >↑in the evening<=I don’t like during the day I don’t

25 **oo ongelmaa eikä oo sillonkaa vielä ku mä töistä mee' niin?**

have problem neither have then+even still when I from+work go so

have problems and neither then still when I come from work so?

26 **.hhh (0.2) siinä syönnis ei oo ongelmaa mut se iltapala on**

in+that eating no is problem but that supper is

.hhh (0.2) there is no problem with eating but the supper is

27 **semmone, (0.3) kompastus°kivi.°**

that+kind+of stumbling+block

kind of a, (0.3) stumbling °block.°

28 (1.1)

29 N: **joo:.**

yes

ye:s.

30 (1.0)

31 B: **°ajatuksen tasolla.=**

thought’s level

°in principle.°=

32 N: **=hirveä[n hyvä m?]**

awfully good

= reall[y good m?]

33 C: **[nii (---)]**

yeah

[yeah (---)]

34 **(0.7)**

35 N: **hirveän hyvä ehdotus,**

awfully good suggestion

really good suggestion,

((Continues))

Extract 4

Group’s perspective (Group 2)

1 N: **Oikei' hyviä konsteja.**

really good tips

Really good tips.

2 N: **Sait kolme pistettä.**

you+got three points

You got three points.

3 N: **[Haluaako] joku lisätä tähä' vielä, (.) jonku asian.**

want+Q somebody add to+this still some thing

[Does somebody want] to add something more, (.) to this issue.

4 ?: **[↑mm:?]**

5 (4.1)

6 A: **No= ei muutaku [(sen-)]**

well no else+than that

Well=nothing more than [that-]

7 N: **[stres]sihän vaikut- (.) ↑ni?**

tress+CLT yeah

[As we know] stress affect- (.) ↑yeah?

8 A: **sem[most- (.) e-] ajattelin että siis sillon=kun tuntuu oikein**

that I+thought that so then when feel+0 really

Th[at- (.) e-] I thought that when=it feels really

9 N: **[>sano vaan.<]**

say PTCL

[>Go ahead.<]

10 A: **>tympeeltä nii,< (0.4) mää ainakii yritän keksiä jotakin semmosta**

dull so I at+least try invent something that+kind+of

>stressful,< (0.4) I at least try to come up with something

11 A: **tekemistä >mistä mää< tykkään.**

activity that I like

to do >that I< like.

12 (1.0) ((Continues))

Nutritionist’s perspective

1 N: **Oikei' hyviä konsteja.**

really good tips

Really good tips.

2 N: **Sait kolme pistettä.**

you+got three points

You got three points.

3 N: **Haluaako joku lisä[tä tähä'] vielä, (.) jonku asian.**

want+Q somebody add to+this still some thing

Does somebody want to [add some]thing more, (.) to this issue.

4 ?: **[(-)]**

5 (4.7)

6 N: **Stressi[hän vaikut-]**

stress+CLT

As we kn[ow stress affect-]

7 B: **[No=ei muutaku,]**

well no else+than

[Well=nothing more than,]

8 (.)

9 N: ↑ni?

yeah

↑yeah?

10 (0.5)

11 N: **>Sano vaan.<=**

say PTCL

>Go ahead.<=

12 B: **=(Et se-)} (0.5) ajattelin että siis sillon=kun tuntuu oikein**

that I+thought that so then when feel+0 really

That- (.) e- I thought that when=it feels really

13 B: **>tympeeltä nii,< (0.4) mää ainakii yritän keksiä jotakin semmosta**

dull so I at+least try invent something that+kind+of

>stressful,< (0.4) I at least try to come up with something

14 B: **tekemistä >mistä mää< tykkään.**

activity that I like

to do >that I< like.

((Continues))
